# Detection of Metallothionein in Javanese Medaka (*Oryzias javanicus*), Using a scFv-Immobilized Protein Chip

**DOI:** 10.3390/s18041069

**Published:** 2018-04-02

**Authors:** Euiyeon Lee, Hyunjin Jeon, Chungwon Kang, Seonock Woo, Seungshic Yum, Youngeun Kwon

**Affiliations:** 1 Department of Biomedical Engineering, Dongguk University, Seoul 04620, Korea; dmldysl@gmail.com (E.L.); 9212jhj@gmail.com (H.J.); iu8974@gmail.com (C.K.); 2 Faculty of Marine Environmental Science, University of Science and Technology (UST), Geoje 53201, Korea; cwoo@kiost.ac.kr (S.W.); syum@kiost.ac.kr (S.Y.); 3 South Sea Environment Research Center, Korea Institute of Ocean Science and Technology (KIOST), Geoje 53201, Korea

**Keywords:** metallothionein, single-chain fragment of variable region (scFv), surface plasmon resonance (SPR), biomarker, sensor chip

## Abstract

Environmental pollution by various industrial chemicals and biological agents poses serious risks to human health. Especially, marine contamination by potentially toxic elements (PTEs) has become a global concern in recent years. Many efforts have been undertaken to monitor the PTE contamination of the aquatic environment. However, there are few approaches available to assess the PTE exposure of aquatic organisms. In this research, we developed a strategy to evaluate the heavy metal exposure of marine organisms, by measuring the expression levels of metallothionein protein derived from *Oryzias javanicus* (OjaMT). OjaMT is a biomarker of heavy metal exposure because the expression level increases upon heavy metal exposure. The developed assay is based on a real-time, label-free surface plasmon resonance (SPR) measurement. Anti-OjaMT antibody and anti-OjaMT single-chain fragment of variable region (scFv) were used as detection probes. Two types of SPR sensor chips were fabricated, by immobilizing antibody or Cys3-tagged scFv (scFv-Cys3) in a controlled orientation and were tested for in situ label-free OjaMT detection. Compared to the antibody-presenting sensor chips, the scFv-presenting sensor chips showed improved performance, displaying enhanced sensitivity and enabling semi-quantitative detection. The portable SPR system combined with scFv-immobilized sensor chips is expected to provide an excellent point-of-care testing system that can monitor target biomarkers in real time.

## 1. Introduction

Environmental pollution by various industrial chemicals and biological agents poses serious risks to human health. Especially, marine contamination by PTE has become a global concern in recent years [1,2]. Many efforts have been conducted to monitor PTE contaminants in the aquatic environment, in order to gauge the damage and trace the source of contamination, which has primarily involved the direct chemical analysis of aquatic samples [3,4]. However, chemical analysis may be insufficient for evaluating the impact of the pollutant on the aquatic organisms. In assessing the risks of environmental pollutants, it is important to understand the damages done to organisms, such as alterations in molecular, cellular, and physiological processes, occurring within an organism, as outcomes of pollutant exposure [5,6,7]. Therefore, the measurement of biomarkers of heavy metal exposure may offer an alternative to conventional chemical analysis. Changes in biomarkers of exposure can provide quantitative as well as qualitative estimates of exposure to various contaminants [8,9].

The induction of metallothionein (MT) synthesis by heavy metals has been demonstrated in numerous species (e.g., mollusks, crustaceans, annelids), prompting suggestions of MT concentrations in organisms as potential biomarkers of heavy metal exposure [10,11,12]. MTs are low-molecular-weight range proteins of 6–7 kDa with unusually high (20–30%) cysteine contents and involved in metal detoxification and homeostasis in living organisms, by coordinating to metal ions with high affinity. In the previous study, significantly higher mRNA levels of *OjaMT* were detected in the liver tissue of *O. javanicus* after 24 h exposure of heavy metals at 0.1–100 μg/mL level [12].

Traditionally, the expression levels of protein biomarkers have been determined indirectly by measuring changes in messenger RNA (mRNA) levels [13]. However, many studies have reported that the correlation between mRNA and protein expression levels is relatively inconsistent [14,15,16]. Alternatively, a few groups have reported the use of enzyme-linked immunosorbent assay (ELISA) for quantitative analysis of MT protein [17,18]. These studies have shown that it is important to quantify protein biomarkers directly to obtain more relevant information. Label-free biosensors are particularly useful tools for the quick detection in crude samples. The surface plasmon resonance (SPR)-based biosensor is a representative label-free technique, capable of detecting low concentrations of biomaterials within a rapid response time [19,20].

In this paper, we developed an approach to monitor the heavy metal contamination of marine ecosystems via detection of MT proteins using SPR. We fabricated sensor chips tailored to present anti-*Oryzias javanicus* MT (anti-OjaMT) single-chain fragment of variable region (scFv) or anti-OjaMT monoclonal antibody (mAb) and evaluated the performance of these sensor chips in OjaMT detection. We also attempted to establish a platform enabling point-of-care testing (POCT) by utilizing portable SPR.

## 2. Materials and Methods

### 2.1. General Procedures

A plasmid DNA Mini-Prep kit and all restriction enzymes were purchased from New England Biolabs (Beverly, MA, USA) and Elpis Biotech (Daejeon, Korea). The polymerase chain reaction (PCR) kit and gel extraction product kit came from Real Biotech Corp (Taipei, Taiwan). All oligomers were commercially synthesized by Bioneer (Daejeon, Korea). General chemicals were product of Sigma Aldrich (St. Louis, MO, USA) in the best grade available. The scFv-Cys3 gene was cloned in the bacterial expression vector pET-28a(+). We used this pET-28a(+)-scFv-Cys3 construct to express a recombinant scFv-Cys3 in *Escherichia coli*. The expression vector and Rosetta (DE3) were purchased from Novagen (Madison, WI, USA). Isopropyl-β-d-thiogalactopyranoside (IPTG) was obtained from Gold Biotechnology (St. Louis, MO, USA). Protein samples were analyzed on 12% polyacrylamide gels and stained with Coomassie Brilliant Blue R250. The gold chip was procured from MiCo NanoBioSys. SPR was performed using a MiCo SPR nano-instrument from MiCo NanoBioSys, using the gold sensor chip.

### 2.2. Purification of Anti-OjaMT mAbs

The mAbs in serum-free hybridoma supernatants were passed through a 0.22 µm filter and applied to a Protein G Sepharose column purchased from Abcam (Cambridge, UK). After washing with 1× PBS, the bound mAb was eluted with 0.2 M glycine, pH 2.0, neutralized with 1 M Tris, pH 8.0, and dialyzed against 1× PBS containing 0.02% (*w*/*v*) sodium azide. The purity of the mAb preparations was determined by sodium dodecyl sulfate-polyacrylamide gel electrophoresis (SDS-PAGE), using a 12% SDS-PAGE gel.

### 2.3. Expression and Purification of Recombinant OjaMT

*E. coli* Rosetta (DE3) cells were transformed with OjaMT cloned in pRSET-C [12]. The transformants were grown to an OD600 nm of 0.6 at 37 °C in shake flasks containing 200 mL of Luria-Bertani (LB) medium (0.5% yeast extract, 1.0% tryptophan, and 1.0% NaCl) with 50 µg/mL ampicillin. The expression was induced by adding IPTG to a final concentration of 0.3 mM. The transformed cells were further grown overnight at 18 °C. The cells were harvested by centrifugation at 3000 rpm for 30 min. The cell pellets were re-suspended in lysis buffer (50 mM NaH_2_PO_4_, 300 mM NaCl, 10 mM imidazole, 1 mM phenylmethylsulfonyl fluoride (PMSF) at pH 8.0) and lysed by sonication. The solubilized protein was separated by centrifugation at 15,000 rpm/4 °C for 30 min. The supernatant was incubated with Ni-NTA resin at 4 °C for 2 h and then applied to the column. The His-tagged recombinant OjaMTs were eluted with native elution buffer (50 mM NaH_2_PO_4_, 300 mM NaCl, and 250 mM imidazole at pH 8.0). The eluted recombinant OjaMTs were dialyzed against PBS at 4 °C to remove imidazole.

### 2.4. Immunoblot Analysis

Purified OjaMT was separated by SDS-PAGE and transferred to a 0.45 µm polyvinylidene fluoride (PVDF) membrane purchased from Millipore (Burlington, MA, USA). This was blocked with 10% (*w*/*v*) skim milk powder in TBS, and the immobilized proteins were detected with anti-OjaMT mAb and a secondary goat anti-mouse IgG HRP conjugate obtained from Bethyl (Montgomery, AL, USA), followed by detection with PicoEPD Western Reagent purchased from Elpis Biotech (Daejeon, Korea).

### 2.5. Isolation of the Variable Heavy and Light Chain (V_H_ and V_L_)

Total RNA was extracted from the OjaMT hybridoma cells (Youngin Frontier, Seoul, Korea) using TRI Reagent^®^. RNA (1 µg) was treated with DNaseΙ and reverse-transcribed into complementary DNA (cDNA) for use as a template for PCR. The reverse transcription was performed at 37 °C for 2 h in a reaction volume of 20 µL containing the following: 1 µL of specific primer (80 µM), 10× Maloney murine leukemia virus (M-MLV) reverse transcription buffer, sterile water (RNase-free, 14 µL), deoxyribonucleotide triphosphates (dNTPs) (2 mM each), RNase inhibitor (40 units/µL), and M-MLV reverse transcriptase (200 units/µL). Reverse transcriptase activity was stopped by heating samples at 72 °C for 10 min. Heavy-chain cDNA was obtained using MuIgG3-Fwd (heavy chain) primer, 5′-CTG GAC AGG GCT CCA TAG TTC CA-3′, and light chain cDNA was obtained MuCK-Fwd (light chain) primer: 5′-CTC ATT CCT GTT GAA GCT CTT GAC-3′. The resulting cDNA was subjected to PCR using the primer set of MuJH1For, 5′-TGA GGA GAC GGT GAC CGT GGT CCC-3′, and MuVH4/6Back, 5′-GAG GTY CAG CTG CAR CAR TCT GG-3′, to amplify the *V_H_* gene and the primer set of MuJK1For, 5′-TTT GAT TTC CAG CTT GGT GCC TCC-3′, and MuVK1Back, 5′-GAC ATT GTG ATG WCA CAG TCT CC-3′, to amplify *V_L_* gene.

### 2.6. Construction of Cys3-Tagged scFv (scFv-Cys3)

**Figure 1:**
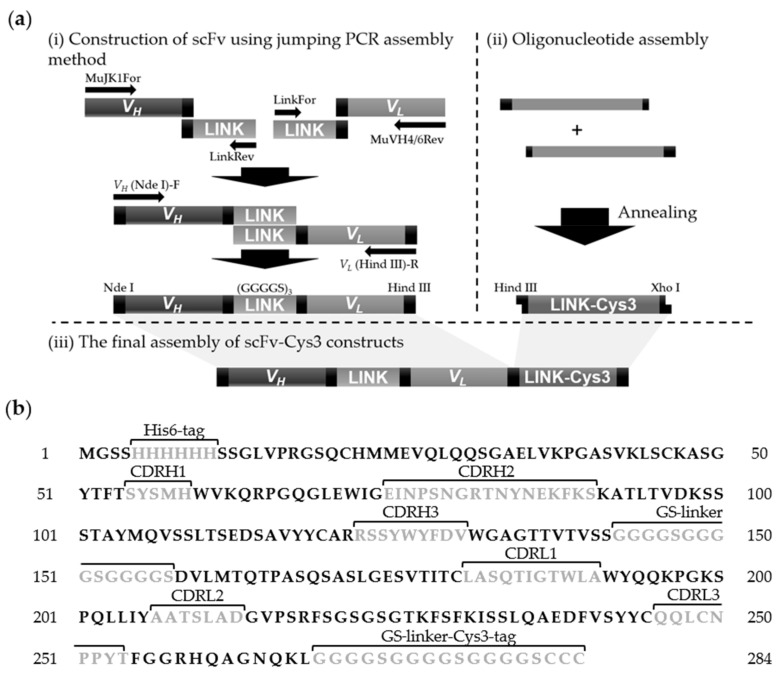
Preparation of a recombinant single chain antibody (scFv) construct recognizing *Oryzias-javanicus*-derived metallothionein (OjaMT). (**a**) The recombinant anti-OjaMT scFv is constructed by splicing overlap extension where the two genes overlap via the (GGGGS)_3_ (GS-linker). (**b**) Amino acid sequence of the *V_H_* and *V_L_* chains are shown. The complementarity-determining regions (CDRs) of variable domains are indicated in gray. [*V_H_*: 119 amino acids, *V_L_*: 107 amino acids, (GGGGS)_3_ (GS-linker): 15 amino acids].

A linker1 and linker2 gene coding (GGGGS)_3_ (GS-linker) were prepared by PCR amplification of GS-linker gene (GGT GGC GGT GGC TCG GGC GGT GGT GGG TCG GGT GGC GGC GGA TCT) by using the primer sets of LinkFor,5′-AGA TCC GCC GCC AC-3′, RevMuJH1For-2, 5′-CGG TCA CCG TCT CCT CAG GTG GCG GTG GCT C-3′, RevMuVK2 Back-2, 5′-CTG TTG CAG CTG AAC CTC AGA TCC GCC GCC AC-3′, and LinkRev, which was 5′-GGT GGC GGT GGC T-3′, respectively. The linker1 gene was annealed to *V_H_* and the annealed gene product was used as a template for PCR amplification using a primer set of MuJK1For, 5′-TTT GAT TTC CAG CTT GGT GCC TCC-3′, and LinkRev, 5′-GGT GGC GGT GGC T-3′, to produce *V_H_*-linker (Figure 1a). The linker2 gene was annealed to *V_L_* fragments and the annealed gene product was used as a template for PCR amplification using a primer set of LinkFor, 5′-AGA TCC GCC GCC AC-3′, and MuVH4/6Rev, 5′-GAG GTY CAG GTC CAR CAR TCT GG-3′, to generate linker-*V_L_*.

In the final assembly reaction, the *V_H_*-linker and linker-*V_L_* genes were annealed with each other and the annealed product was used as a template for jumping PCR using a primer set of *V_H_*, (Nde I)-F, 5′-CAT ATG GAG GTT CAG CTG CAG CAG T-3′, and *V_L_*, (Hind III)-R, 5′-AAG CTT TTG ATT TCC AGC TTG GTG CCT-3′, to generate scFv. The recombinant scFv fragment (*V_H_*-linker-*V_L_*) was then inserted into a bacterial expression vector pET-28a(+) using Nde I/Hind III restriction sites to make pJDH033. The GS-linker-Cys3 fragment was prepared by complementary oligonucleotide synthesis to generate the sequence: 5′-AGC TTG GTG GCG GTG GCT CGG GCG GTG GTG GGT CGG GTG GCG GCG GAT CTT GTT GCT GTT GAC-3′. This double strand DNA was inserted into the Hind III/Xho I sites of pJDH033 to produce pJDH043.

### 2.7. Expression and Purification of Recombinant scFv

*E. coli* Rosetta (DE3) cells were transformed with pJDH043. The transformants were grown to an OD600 nm of 0.6 at 37 °C in shake flasks containing 200 mL of LB medium with 50 µg/mL kanamycin. The expression was induced by adding IPTG to a final concentration of 0.3 mM. The transformed cells were further grown overnight at 18 °C. The cells were harvested by centrifugation at 3000 rpm for 30 min. The cell pellets were re-suspended in lysis buffer (50 mM NaH_2_PO_4_, 300 mM NaCl, 10 mM imidazole, 1 mM PMSF at pH 8.0) and lysed by sonication. The soluble and insoluble fractions were then separated by centrifugation at 15,000 rpm/4 °C for 30 min. Lysis buffer containing 8 M urea was added to solubilize the pellet, and the solution was incubated with Ni-NTA resin at 4 °C, with stirring for 3 h. The loaded column was washed stepwise with 50 mL of 4, 3, 2, 1, and 0.5 M urea, and finally with 50 mL of wash buffer (50 mM NaH_2_PO_4_, 300 mM NaCl, and 20 mM imidazole at pH 8.0). The His6-tagged recombinant scFv’s were eluted with native elution buffer (50 mM NaH_2_PO_4_, 300 mM NaCl, and 250 mM imidazole at pH 8.0). The eluted recombinant scFv’s were dialyzed against PBS at 4 °C to remove imidazole. Protein samples were analyzed by denaturing SDS-PAGE. The gel was dyed with Coomassie Blue staining solution. The protein concentration was determined by the Bradford method, with BSA as the standard.

### 2.8. Experimental Fish

We used 6–9-month-old Javanese medaka (*Oryzias javanicus*, 2.4–2.8 mm; weight: 0.13–0.17 g) which were cultured in the KIOST (Geoje, Korea). Twenty fish were placed in a 3 L tank at 25 ± 1 °C in natural seawater filtered through serially connected filters of different pore sizes (100, 10, and 1 μm) with a controlled normal photo-regimen (16 h light–8 h dark). The fish were fed with *Artemia* sp. naupii once a day.

### 2.9. CdCl_2_ Exposure and Sampling (Heavy Metal Exposure and Sampling)

Four groups of five fishes were prepared. The fish were transferred to 1 L beakers containing 0.8 L of seawater. After acclimation for 48 h without food, the experimental groups were exposed to 0.1, 1, and 10 mg/L CdCl_2_ (Sigma-Aldrich Ltd.) for 24 h, respectively. The exposure concentrations were selected after consideration of the 24 h LC_50_ (the median lethal concentrations) value for Javanese medaka (44.25 mg/L) [21]. A group of untreated fish was prepared as a control. Liver tissues were collected from each group after rendering fish unconscious with cold shock. Total protein was extracted from the fish livers with Pro-Prep Protein Extraction Solution (Intron Biotechnology, Sungnam, Korea), according to the manufacturer’s instructions.

### 2.10. SPR Analysis

The gold chip surface was cleaned with concentrated piranha solution (H_2_SO_4_:H_2_O_2_ = 7:3 *v*/*v*) and thoroughly rinsed with ethanol and deionized water. All SPR spectroscopy experiments were performed with the pre-cleaned gold chip on a MiCo SPR nano-device at room temperature using the PBS buffer as a running solution unless indicated otherwise. The SPR sensor chip presenting antibodies was prepared by coating the protein G-Cys3 (0.1 mg/mL, 10 µL/min for 15 min) onto the gold substrate and capturing the anti-OjaMT mAb site-specifically (50 µg/mL, at a flow rate of 10 µL/min for 15 min). Unconjugated antibody was washed and blocked by flowing PBS and PEG–thiol solution (0.1 mM, 10 µL/min for 10 min), sequentially. The SPR sensor chip presenting scFv was prepared by coating the scFv-Cys3 (50 µg/mL, 10 µL/min for 15 min) onto the gold substrate. The scFv-presenting sensor chip was washed with PBS and blocked with PEG–thiol solution (0.1 mM, 10 µL/min for 10 min). After the immobilization of probes, the OjaMT (5–30 µg/mL, in PBS) was flowed over the sensor chip at 10 µL/min for 10 min. The sensor chip was washed with PBS to remove unbound OjaMT, and the difference in RU was recorded.

## 3. Results and Discussion

### 3.1. Preparation of OjaMT Detection Probes

Anti-OjaMT mAbs are obtained from the culture media of customized hybridoma cells. The mAbs are purified by affinity chromatography using a protein G column, according to a standard protocol [22]. The purified mAbs are treated with a sample buffer containing a reducing agent, β-mercaptoethanol, to separate the light (*V_L_*) and heavy (*V_H_*) chains connected via disulfide bonds and are then analyzed by gel electrophoresis. The gel was stained using Coomassie Brilliant Blue stain, to reveal the presence of both chains (Figure S1a). The binding activity and specificity of the purified mAb were tested by immunoblotting. Western blot analysis was performed using the purified mAb as the primary antibody for detection of OjaMT. The HRP-conjugated goat anti-mouse IgG was used as a secondary antibody, considering that the hybridoma cells were derived from the mouse. Immunoblot analysis showed that the purified mAb retains a specific binding affinity for the target OjaMT protein (Figure S1b).

Next, we prepared scFv that recognize OjaMT, by linking the *V_H_* and *V_L_* domains through a linker of a single polypeptide for enhanced affinity, stability, and expression levels. The *V_H_* and *V_L_* chains are the smallest Ab fragments that contain the entire antigen recognition sites. As there is no preference on the relative position of each chain, we followed a conventional design to place the *V_H_* chain on the N-terminus and *V_L_* on the C-terminus, respectively. Linker peptides were introduced to produce single-chain peptides with minimal steric interference between two heterodimeric variable fragments. The most widely used (GGGGS)_3_ (GS-linker) was adopted in our experiments (Figure 1a). We constructed the scFv using a previously reported jumping PCR assembly method [23]. The construction of scFv was confirmed by DNA agarose gel electrophoresis and sequencing (Figure S2a and Figure 1b). The scFv was then modified to carry both His6-tag and Cys3-tag. The His6-tag was introduced for purification, and the Cys3-tag was for the immobilization on a gold substrate. The thiol group of cysteine can be used as an effective tool for oriented immobilization of proteins because it bonds strongly with gold substrates to form a self-assembled monolayer [24]. The expression and purification of recombinant scFv were confirmed by gel electrophoresis analysis (Figure S2b).

### 3.2. Preparation of mAb Sensor Chip and In Situ Real-Time Detection of OjaMT

**Figure 2:**
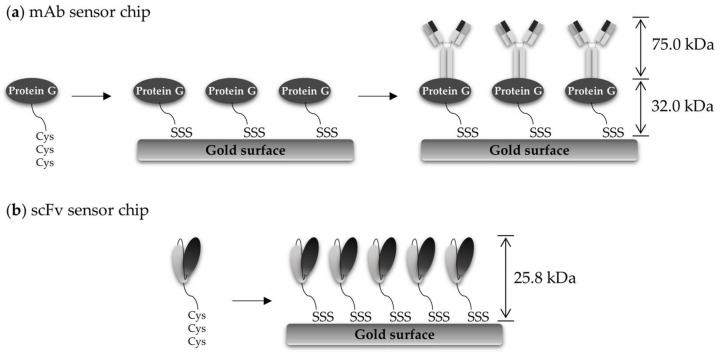
A schematic diagram of probe immobilization on surface plasmon resonance (SPR) chip for immuno-sensing of metallothionein derived from *Oryzias javanicus* (OjaMT). (**a**) Monoclonal antibodies were immobilized on gold surface via protein G-mediated interaction. (**b**) Recombinant scFv-Cys3 was directly immobilized on gold surface via thiol–gold interaction.

In order to fabricate the SPR sensor chips, the probes need to be immobilized to the gold substrate in a functional form. The affinity, orientation, and stability of probe proteins are often influenced by immobilization strategies. In particular, the binding site of the probe protein should be oriented to recognize soluble targets. We first prepared functional SPR sensor chips by selectively immobilizing mAbs using a portable SPR system from MiCo NanoBioSys (Sungnam, Korea) (Figure 2a). The gold-coated glass slide was coated with Cys3-tagged protein G (protein G-Cys3) (0.1 mg/mL), which binds to the constant region of the heavy chain antibody, by injecting the solution through the flow cells at a flow rate of 10 µL/min for 15 min. Then, the protein-G-coated slide was treated with the anti-OjaMT mAb (50 µg/mL) for site-specific immobilization. The binding of protein G and immobilization of anti-OjaMT mAb were monitored using SPR sensorgrams. The SPR sensorgram shows the refractive index change upon immobilization of protein G (∆RU = 2300) and mAb (∆RU = 2100). 

**Figure 3:**
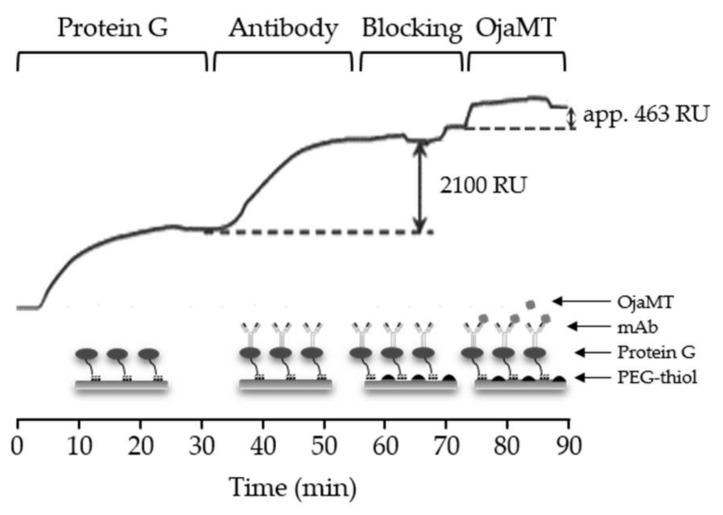
OjaMT detection using SPR chip presenting anti-OjaMT monoclonal antibody (mAb). An antibody (50 μg/mL) was immobilized on a gold surface, and OjaMT (30 μg/mL) was then flowed at a rate 10 μL/min for 10 min. RU stands for response unit.

The SPR sensor chip was then used for real-time detection of target OjaMT proteins in situ. Solutions containing PEG–SH (100 µM), OjaMT (30 µg/mL), and phosphate-buffered saline (PBS) were sequentially injected into the flow cell of the sensor chip at a flow rate of 10 µL/min. The PEG–SH solution was used as a blocking buffer to prevent any non-specific binding and PBS was used as a wash buffer to remove unbound proteins. The changes in the refractive index were monitored continuously in real time using the SPR. The SPR sensorgram showed the refractive index change upon binding of OjaMT was ~500 RU and the signal did not change upon the PBS wash (Figure 3). While the prepared mAb SPR sensor chip showed selective binding of the target OjaMT, it requires two protein immobilization steps for sensor chip fabrication. In addition, the large-sized mAb and protein G limit the density of the probe proteins, which can adversely impact the sensitivity.

### 3.3. Preparation of scFv Sensor Chip and In Situ Real-Time Detection of OjaMT 

We then generated anti-OjaMT scFv-immobilized SPR sensor chips. Anti-OjaMT scFv was genetically engineered to contain Cys3 residues at the C-terminus. scFv-Cys3 was stably expressed and purified. Recombinant scFv antibodies were immobilized onto a gold substrate in a specific orientation via stable multivalent sulfur and gold interactions, in a single-step process (Figure 2b). 

**Figure 4:**
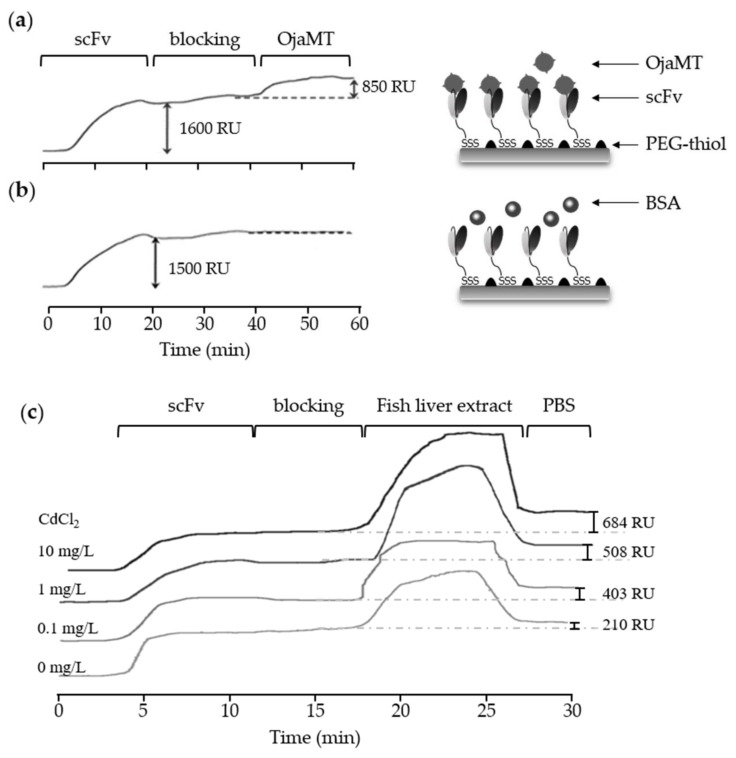
OjaMT detection using SPR chip presenting recombinant anti-OjaMT scFv. (**a**) Recombinant scFv (50 μg/mL) was immobilized on a gold surface, and antigen-OjaMT (30 μg/mL) was then flowed at a rate 10 μL/min for 10 min. (**b**) The immobilized recombinant scFv fragments (50 μg/mL) showed non-specific binding with BSA, control protein (30 μg/mL). (**c**) The presence of OjaMT in heavy metal contaminated fish liver was monitored using a scFv-immobilized sensor chip. All the contaminated samples showed increased ΔRU, suggesting the increase in OjaMT level.

The portable SPR system was used to monitor the in situ generation of the scFv SPR sensor chip and target binding (Figure 4). For detection of OjaMT, scFv-Cys3 was injected over the flow cells of the pre-cleaned gold chip. The immobilized recombinant scFv quickly reached the saturation level with ΔRU of 1500–1600. A sensor chip presenting scFv was blocked with PEG–SH solution to prevent non-specific binding, flowed with OjaMT, and washed with PBS, sequentially. The captured OjaMT increased SPR signal approximately 850 RU (Figure 4a). The BSA solution was used for negative control and did show significant binding (<80 RU) to scFv probes (Figure 4b). The scFv presenting sensor chip showed higher reactivity to OjaMT binding compared to the antibody presenting sensor chip. The scFv presenting chip can also be prepared more quickly than antibody chips because the precoating with protein G-Cys3 is not required. Furthermore, scFv molecules smaller than mAbs can be immobilized at high density on the chip surface. Our approach produced a densely distributed scFv on a gold surface to exhibit improved sensitivity to the OjaMT binding compared to antibody presenting chips.

### 3.4. Detection of OjaMT in Liver Samples of Heavy Metal Contaminated Oryzias javanicus Using scFv Sensor Chips

We finally explored the capability of the developed scFv sensor chip in detecting the OjaMT in crude fish samples. We first tested the detection capability of the scFv sensor chip and the portable SPR system by using the purified OjaMT samples in concentrations ranging from 5 to 30 µg/mL, which is compatible with the range of OjaMT in crude fish samples [25]. The solution of purified OjaMT was injected onto the scFv sensor chip, and ΔRU was recoded (Figure S3). The system was able to report the presence of purified OjaMT in all three concentrations. We then prepared protein extracts from fish livers exposed to cadmium in concentrations of 0, 0.1, 1, and 10 mg/L. Each sample was analyzed using the scFv sensor chip. The binding of OjaMT was recoded as ΔRU. All three cadmium-treated samples showed higher ΔRU compared to the non-treated control. The measured ΔRU values increased with the heavy metal concentration, suggesting that the amount of MT protein increased. The non-treated control sample also showed considerable binding probably due to the basal level of MT proteins as well as non-specific binding of random proteins. The results suggest that the scFv sensor chips combined with the portable SPR system has great potential for use as a real-time detection method to monitor heavy metal contamination in aquatic systems. 

## 4. Conclusions

In this paper, we described an approach to monitor the heavy metal exposure of the marine organism *Oryzias javanicus*, by measuring a biomarker protein, metallothionein, in real time. The approach is based on a portable SPR system, which allows label-free detection of target proteins. For this work, we prepared two types of detection probes, namely, anti-OjaMT monoclonal antibody and anti-OjaMT scFv. The anti-OjaMT monoclonal antibody was harvested using hybridoma cells, and scFv was recombinantly prepared based on a jumping PCR assembly method. The recombinant scFv was modified to carry Cys3-tag for site-directed immobilization. The sensor chips were made by immobilizing anti-OjaMT monoclonal antibody or anti-OjaMT scFv and were tested for in situ label-free OjaMT detection. The scFv-presenting sensor chips showed improved performance compared to antibody-presenting sensor chips. The sensor chip was fabricated by short immobilization steps, without the presence of the large linker protein, protein G, which can mediate unwanted interactions. The scFv sensor chips were finally used for the detection of OjaMT in fish liver from heavy metal contaminated *Oryzias javanicus.* We were able to distinguish the contaminated samples from non-treated control. The developed system provides a generic platform to monitor target biomarkers in real time for environmental contamination monitoring.

## Supplementary Materials

The following are available online at http://www.mdpi.com/1424-8220/18/4/1069/s1. Figure S1. Production and characterization of anti-OjaMT monoclonal antibody (mAb). (a) Purification of anti-OjaMT mAb was analyzed by SDS-PAGE. Lane 1: hybridoma media; Lane 2: protein G column flow through; Lanes 3 and 4: wash fraction; Lane 5: elution; M: protein weight marker. (b) SDS-PAGE (left) and western blot analysis using the anti-OjaMT mAb as the primary Ab (right). Lane 1: control protein (maltose binding protein (MBP)); Lane 2: OjaMT; M: protein weight marker. Figure S2. Preparation of the recombinant anti-OjaMT scFv construct. (A) The recombinant anti-OjaMT scFv antibody were analyzed by DNA electrophoresis. Lane 1: heavy chain; Lane 2: linker assembled heavy chain; Lane 3: light chain; Lane 4: linker assembled light chain; Lane 5: final assembly of recombinant anti-OjaMT scFv constructs; M: DNA ladder. (C) Purification of anti-OjaMT scFv was analyzed by SDS-PAGE. Lane 1: before refolding sample; Lane 2: Ni-column flow through; Lanes 3 and 4: wash fraction; Lane 5: the final refolded recombinant anti-OjaMT scFv-Cys3; M: molecular weight marker. Figure S3. OjaMT detection using SPR chip immobilized with recombinant anti-OjaMT scFv. Sensitivity of scFv-immobilized sensor chip was confirmed through the change of RU value after OjaMT was exposed in the concentration range of 5 μg/mL to 30 μg/mL.
