# Supplementary material for: Detection of Metallothionein in Javanese Medaka (Oryzias javanicus), Using a scFv-Immobilized Protein Chip
Source: Sensors (Basel). 2018 Apr 2;18(4):1069. doi: 10.3390/s18041069 (PMC5948571; doi:10.3390/s18041069)
Supplement: Supplementary file 1 [file sensors-18-01069-s001.pdf]

Detection of a heavy metal exposure biomarker, metallothionein in Javanese medaka *(Oryzias javanicus)* using scFv-immobilized protein chip

Euiyeon Lee [1,†], Hyunjin Jeon [1,†], Chungwon Kang [1], Seonock Woo [2,3], Seungshic Yum [2,3] and Youngeun Kwon [1,*]

[1] Department of Biomedical Engineering, Dongguk University, Seoul 04620, Korea; dmldysl@gmail.com, iu8974@gmail.com, ykwon@dongguk.edu
[2] South Sea Environment Research Center, Korea Institute of Ocean Science and Technology (KIOST), Geoje 53201, Korea; cwoo@kiost.ac.kr, syum@kiost.ac.kr
[3] Faculty of Marine Environmental Science, University of Science and Technology (UST), Geoje 53201, Korea; cwoo@kiost.ac.kr, syum@kiost.ac.kr
† These two authors contributed equally.
* Correspondence: ykwon@dongguk.edu; Tel.: +82-31-961-5151

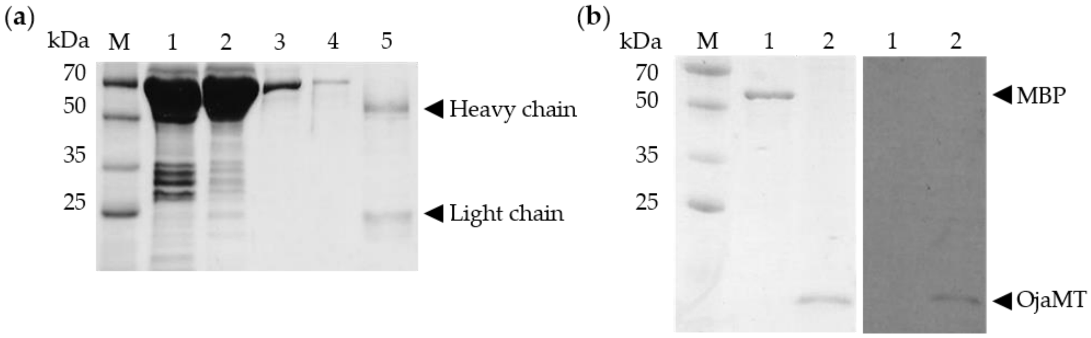

Figure S1. Production and characterization of anti-OjaMT monoclonal antibody (mAb). (**a**) Purification of anti-OjaMT mAb was analyzed by SDS-PAGE. Lane 1, hybridoma media; lane 2, protein G column flow through; lane 3 and 4, wash fraction; lane 5, elution; M, protein weight marker. (**b**) SDS-PAGE (left) and western blot analysis using the anti-OjaMT mAb as the primary Ab (right). Lane 1, control protein (maltose binding protein, MBP); lane 2, OjaMT; M, protein weight marker.

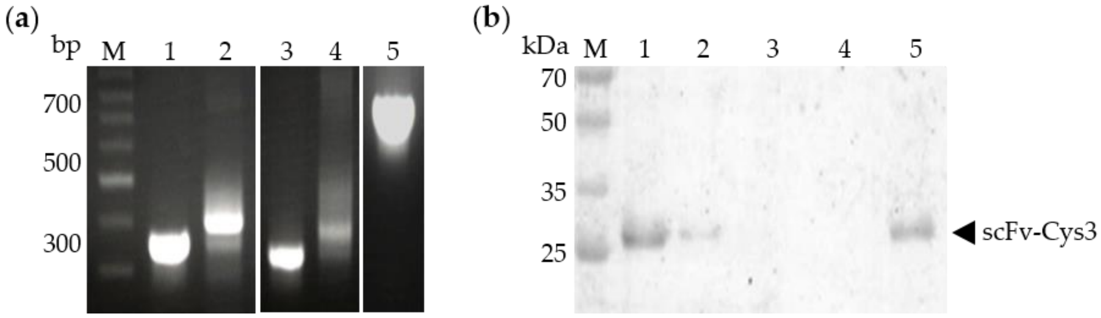

Figure S2. Preparation of the recombinant anti-OjaMT scFv construct. (A) The recombinant anti-OjaMT scFv antibody were analyzed by DNA electrophoresis. Lane 1, heavy chain; lane 2, linker assembled heavy chain; lane 3, light chain; lane 4, linker assembled light chain; lane 5, final assembly of recombinant anti-OjaMT scFv constructs; M, DNA ladder. (C) Purification of anti-OjaMT scFv was analyzed by SDS-PAGE. Lane 1, before refolding sample; lane 2, Ni-column flow through; lane 3 and 4, wash fraction; lane 5, the final refolded recombinant anti-OjaMT scFv-Cys3; M, molecular weight marker.

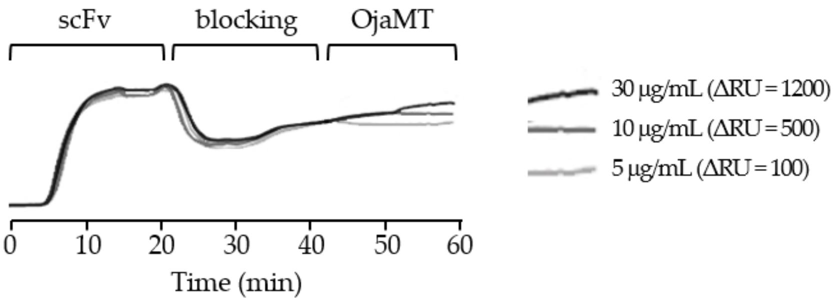

Figure S3. OjaMT detection using SPR chip immobilized with recombinant anti-OjaMT scFv. Sensitivity of scFv-immobilized sensor chip was confirmed through the change of RU value after OjaMT was exposed in the concentration range of 5 μg/mL to 30 μg/mL.
